# Parental Translation into Practice of Healthy Eating and Active Play Messages and the Impact on Childhood Obesity: A Mixed Methods Study

**DOI:** 10.3390/nu10050545

**Published:** 2018-04-27

**Authors:** Alyssa Huxtable, Lynne Millar, Penelope Love, Colin Bell, Jill Whelan

**Affiliations:** 1 School of Health and Social Development, Global Obesity Centre, Deakin University, Geelong 3220, Australia; 2 Australian Health Policy Collaboration, Victoria University, Melbourne 3000, Australia; lynne.millar@vu.edu.au; 3 Australian Institute for Musculoskeletal Science (AIMSS), The University of Melbourne and Western Health, St Albans 3021, Australia; 4 School of Exercise and Nutrition Sciences, Institute for Physical Activity and Nutrition (IPAN), Deakin University, Geelong 3220, Australia; penny.love@deakin.edu.au; 5 School of Medicine, Global Obesity Centre, Deakin University, Geelong 3220, Australia; colin.bell@deakin.edu.au

**Keywords:** childhood obesity, parents, nurse, prevention, maternal and child health

## Abstract

Childhood obesity is a significant health issue worldwide. Modifiable risk factors in early childhood relate to child healthy eating and active play, and are influenced by parents. The aim of the study was two-fold. Firstly, to determine the weight status of children aged between birth and 3.5 years in a rural and remote area of Australia. Secondly, to explore the relationship between child weight status and translation of advice on healthy eating and active play provided to parents by local, nurse-led, Maternal Child Health (MCH) services. Measured anthropometric data (*n* = 438) were provided by MCH services. Semi-structured interviews were conducted with two MCH nurses and 15 parents. Prevalence of overweight/obesity was calculated. Local childhood overweight/obesity prevalence was lower than the national average at age 3.5 years (11.38%; 20%). Parents identified the MCH service as a key source of healthy eating and active play advice and reported mostly following recommendations but struggling with screen time and fussy eating recommendations. We observed a relaxation in parent attitudes towards healthy child behaviours which coincided with a trend towards obesity from 12 months (*p* < 0.001). MCH services provide useful and effective advice to parents but ongoing support is required to prevent obesity later in childhood.

## 1. Introduction

Childhood obesity is a serious public health issue that affects over 41 million children under the age of five years globally [1]. In Australia, 20% of 2–4-year-old children are already experiencing overweight or obesity [2]. Obesity is related to serious physical and mental health issues during childhood and can track into adulthood, with concomitant co-morbidities [3,4]. Although childhood obesity levels are high, prevention is possible through management of risk factors such as consumption of calorie-dense food [5], rapid infant weight gain [6], low levels of physical activity and exceeding screen time guidelines [7]. Some studies have found prolonged breastfeeding to be a protective factor against childhood obesity [8,9]. Promotion of protective behaviours is paramount in obesity prevention efforts, especially in the first 1000 days of life [10].

Parents play a significant role in the development of their children by shaping physical activity and eating behaviours early in childhood [11]. Parental practices including feeding style/control [12], role modelling, using food as a reward [13], and low levels of health literacy [14] have all been associated with child physical activity and eating behaviours linked to weight status. Interventions targeting these parental behaviours have reported wide variation in effectiveness [15]. A range of factors can influence uptake of health promoting behaviours [15], and best practice interventions should consider the individual circumstances of the community [16]. Exploration of the implementation factors related to successful obesity prevention in childhood is ongoing. 

In Victoria, Australia, the Maternal Child Health (MCH) service is a free support service offered to all parents of children from birth to six years. MCH nurses take child measurements, and provide information and advice to parents at key developmental ages guided by the Key Ages and Stages (KAS) framework [17] shortly after childbirth (via home visit), and nine clinic-based consultations (2 weeks, 4 weeks, 8 weeks; 4 months, 8 months, 12 months, 18 months, 2 years and 3.5 years). Child healthy eating and active play recommendations and resources are given to parents at the home visit, 4-month, 12-month and 3.5-year consultations. Recommendations include information on breastfeeding, tummy time, transition to solid foods, quantity and types of food to feed children in the first year of life, family meal times, fussy eating, water and milk as the drink of choice, limiting screen time, and healthy eating and physical activity for toddlers. 

This study is based in Yarriambiack Shire Council, a rural, remote local government area (LGA) in north-west Victoria, Australia with high rates of adult overweight/obesity (63.5%; compared with Victorian State average of 49.8%) [18]. The LGA has a small population (population density 0.9), relatively low levels of cultural diversity (5.9% of the population born overseas compared to the Victorian average of 28.3% [19]), low levels of education (8.3% with Bachelor degree or above compared with 24.3% of Victorians), and is an area of socioeconomic disadvantage (in lowest quintile for Victoria) [20]. There is limited access to fresh fruits and vegetables as there are no dedicated fruits and vegetable stores and only 6 supermarkets spread over 7000 sq kilometres [21]. Some of the medical practitioners in the area offer a bulk-billing system with no out-of-pocket expenses for patients. A Deakin University research project was established in 2015 to investigate the systems level drivers of obesity in the area and implement evidence informed interventions across the life course [21]. The LGA averages 60 births per year [20] and is serviced by two MCH nurses. The area is among the top five LGA’s in Victoria for sustained MCH engagement with 98% of parent-child dyads attending the 3.5 year KAS consultation [22]. 

Given the health consequences of overweight and obesity and the limited published evidence to support sustainable, effective prevention, understanding how parents can effectively translate prevention messages in their home environments is paramount. 

The aim of the study was two-fold. Firstly, to determine the weight status of children aged between birth and 3.5 years in a rural and remote area of Australia. Secondly, to explore the relationship between child weight status and translation of advice on healthy eating and active play provided to parents by local, nurse-led, Maternal Child Health (MCH) services. To answer this, our research questions were:

1. What is the prevalence of overweight/obesity among children aged 0 to 3.5 years in this rural and remote area of Australia?
2. Is there a relationship between weight status and attendance at key MCH consultations where healthy eating and active play were discussed?
3. What influences the application [translation into practice] of healthy eating and active play messages by mothers attending MCH consultations?

## 2. Materials and Methods 

We based this manuscript on the EQUATOR network reporting standards for organisational case studies [23] and qualitative sections were guided by the COREQ published standard for the reporting of qualitative research [24]. Our project was based on a pragmatic worldview where childhood obesity is a result of complex interactions between social, cultural, familial and educational factors [25]. 

### 2.1. Study Design

A case study approach was used to explore factors affecting parental translation of MCH recommendations into practice pertinent to the defined LGA. This approach was chosen to explore the real world experiences of local community members [26] in the raising of healthy children in a complex environment known for high levels of adult overweight and obesity, remoteness and engagement with MCH nurses. 

Data collection was completed with a sequential mixed methods approach [27]. Quantitative anthropometric data were first extracted and analysed to identify local levels of childhood obesity compared with national averages. Parent and nurse interviews followed to explore whether the MCH service supported parents to raise healthy weight children. The following data collection methods were utilised:

- Quantitative anthropometric data analysis describing weight status of children at each of the MCH ‘key ages’;
- Semi-structured parent interviews describing ease or difficulty in following MCH healthy eating and active play recommendations, and;
- Semi-structured MCH nurse interviews describing nurse experiences promoting healthy eating and active play messages to parents.

Ethics approval was obtained from Deakin Faculty of Health for parent and nurse interviews (HEAG-H 02_2017). Ethics exemption was granted for the extraction and analysis of de-identified MCH anthropometric data (DUHREC 2016_399).

### 2.2. Recruitment

Recruitment strategies targeted parents who had engaged in MCH services over a minimum of 12 weeks or three consultations and had at least one child within the MCH age range. For this reason, parents of children aged 12 months–5 years were targeted during recruitment. Recruitment occurred between February and March 2017; flyers were distributed to nine playgroup leaders (listed in the online community directory) with a request to distribute to their networks. Social media and local newspaper advertising strategies were also used. Ethics required interested parents to contact the researchers to volunteer to interview. Recruitment strategies resulted in 21 parents engaged; majority (*n* = 14) recruited by word of mouth through engaged participant networks. Participants were screened for eligibility via phone prior to interview, with five parents excluded [due to pregnancy (*n* = 3), not having a child between the ages of 12 months and 5 years (*n* = 1) and engaging in MCH services outside of the LGA (*n* = 1)]. One parent dropped out prior to interview due to childcare commitments, therefore 15 parents were included within the parent sample. The two MCH nurses in the area agreed to participate prior to project commencement due to the partnership established through the broader research project. All interviewees gave their informed consent to participate in this study prior to interview. As compensation for their time, a $25 supermarket voucher was provided to parents who participated in the study. The nurses did not receive compensation as all interviews were scheduled during their normal work hours. 

### 2.3. Data Collection: Anthropometrics

Anthropometric data collection occurred in November 2016. In addition to ethics exemption, local and state government approval was obtained for the extraction and analysis of de-identified child demographic and anthropometric data recorded by MCH services for all children aged 0–5 years. MCH measurements between November 2011 and November 2016 were extracted manually. Data were collected for the local population of 0–5-year-old children (*n* = 438), which included child birth date, sex, age at each consultation, head circumference, weight and length. 

### 2.4. Data Collection: Interviews

The interview guides were informed by the healthy eating and active play recommendations as mandated by the KAS framework, with additional questions on the number of MCH visits attended and key information sources. Parent and nurse interview guides were pilot tested for coherency prior to interview. 

Key topics covered in parent interviews included number of MCH visits, breastfeeding, transition to solid foods, fussy eating, family meal times, variety in child diet, tummy time, screen time, active play and parent recollection of MCH support/advice given for each of these experiences. Parents were also asked what factors helped or prevented them from implementing advice given by MCH nurses as well as sources of child development information (see Appendix A for parent interview guide). To reduce the potential issue of parents reporting only positive experiences due to close relationships with the MCH nurses, and/or still receiving support through the MCH service, each interview was prefaced with an explanation that the study was not a critique of the MCH nurses/service, but aimed to better understand what impacts parental translation of MCH advice into practice within the busy home environment. 

Parent interviews were completed in two rounds between March and April 2017. Two rounds of interviews were required due to not having met data saturation during the first round. Parent interviews were conducted by the lead author (AH) (*n* = 12 interviews) and the senior author (JW) (*n* = 3 interviews). The interview schedule was structured to reduce variation between interviews. Interviews lasted on average 29 min (range: 13 to 39 min). 

Nurse interviews were conducted in March 2017 by AH and covered healthy eating, active play and child weight/development discussions had with parents; timing of nurse discussions; parent receptivity to healthy eating/active play messages; barriers to nurse discussions; and barriers/enablers to parents implementing advice (see Appendix B for nurse interview guide). 

### 2.5. Data Analysis

Quantitative data were manually entered into Stata Release V 14.0 [28] and checked for missing data and outliers on each variable. Where missing data and outliers were detected, the data were first checked against the hard copy of the data sheet to exclude data entry error. Where there was no entry error, the MCH nurses were contacted for clarification and the database was updated accordingly. The final dataset contained no outliers or non-random missing data. 

Descriptive statistics (*t*-tests, chi square and tests on the equality of proportions, where appropriate) were used to present the demographic characteristics of the sample (gender, age, number of consultations). Age and sex standardized Body Mass Index (BMI-z) scores were calculated using World Health Organization (WHO) growth references then cut-points for childhood overweight/obesity were calculated [29]. The international criteria for overweight and obesity by gender (ages 2 to 18) developed by The International Obesity Taskforce (IOTF) were also used [30]. Average BMI-z scores and proportion of children overweight/obese were calculated for each ‘key age’ as defined by MCH KAS framework. 

Logistic regression was used to test the relationship between children who attended/did not attend the 4-month consultation (where information on healthy eating is provided) and child weight status at the subsequent 12 month and 3.5-year consultation. Child age and sex were controlled for in the model. Logistic regression followed by trend analysis was used to test the difference in prevalence of overweight/obesity at each KAS consultation. 

All interviews were audio-recorded with participant consent and transcribed verbatim by the lead author. Participants were given the option to check their transcript prior to data analysis (*n* = 2 accepted, no changes or omissions were made). Transcripts were imported into NVivo 11 [31] and coded using a priori coding scheme based on the interview schedules. These were: healthy eating (breastfeeding, transition to solids, meal times, fussy eating, core food groups, healthy drinks, information sources), active play (tummy time, outdoor play, screen time, organised activities, information sources) and engagement with the MCH service. Emergent themes ‘relaxed attitudes’ and ‘MCH supportiveness’ were added during analysis. All transcripts were coded by AH, with a 20% sub-sample coded by JW to check for coding consistency. 

### 2.6. Reflexivity and the Role of the Researcher

This mixed method study has a strong qualitative focus, therefore it is important to recognise the role of the researcher in the collection, analysis and interpretation of the data. The lead author (AH) has a sound knowledge base of food and nutrition and the social determinants that influence population health. She has experience engaging with disadvantaged populations through her work with homeless cohorts and other public health projects. AH had no affiliation with the local government area prior to commencing study in 2016, and has not yet experienced parenthood. JW is a mother and has been immersed in the local community for over three years, has developed community trust and has significant experience in design, implementation and evaluation of community based interventions, collaborative efforts, nutrition and food security. 

## 3. Results

### 3.1. Participant Demographics 

Quantitative population data were extracted from the MCH computer records for all children 0–5 years old who had attended the local MCH service between November 2011 and November 2016, resulting in data for 438 children. All interview participants (*n* = 15) had at least one child between the ages of 12 months and 5 years, with nine parents having multiple children within the age group. The majority of parent interviewees were female (one male), and average age was 31 years (range 20–39 years old). Demographic data were not collected for the two MCH nurses to protect confidentiality. 

### 3.2. Quantitative Results 

#### Key Indicator: Anthropometric Results 

Of the 438 children, 46.4% (*n* = 203) were male, 53.6% (*n* = 235) were female (see Table 1). There was no significant difference (*p* > 0.05) in the proportion of males/females. Additional consultations recorded in the MCH database (additional to the 10 KAS consultations), showed the mean number of additional consultations per child was seven (SD 5; range 0–22) and the median was also seven.

MCH consultations of interest within this study were the 4-month, 12-month, 2-year and 3.5-year consultations. Table 1 shows data extracted for these consultations in addition to the 18-month consultation.

Child BMI-z scores were calculated for all children and classified as ‘underweight’, ‘normal weight’, ‘overweight’ or ‘obese’ using WHO growth charts [29]. As there were very few children in the underweight or obese categories, these were collapsed into the normal range and overweight/obese range respectively (Table 1). At the 3.5-year consultation, 88.6% (*n* = 148) of children were of a normal weight, and 11.38% (*n* = 19) were overweight or obese.

At the 2-year consultation 189 (91.3%) of children were in the thinness/normal weight range and 18 (8.7%) in the overweight/obese range using the IOTF weight categories [30]. At the 3.5-year consultation, 141 (84.9%) of children were in the thinness/normal weight range and 25 (15.1%) in the overweight/obese range. Weight status was lower using IOTF cutpoints because the WHO cutpoints are set for an ideal population (Table 1).

Logistic regression followed by trend analysis was used to test the difference in prevalence of overweight/obesity at each of the MCH consultations. The results show that compared to the 12-month consultation, children at the 3.5-year consultation were more than twice as likely to be in the overweight/obese group (OR 2.4; *p* < 0.05) (Table 2). A significant upwards trend in the prevalence of overweight/obesity was noted starting from 12 months (*p* < 0.001; see Table 2).

The weight status of 3.5-year-old children who did or did not attend the 4-month consultation where healthy introduction to solid foods is discussed, was compared. Of the children who did not attend the 4-month consultation, 100% (*n* = 25) were of normal weight status at 3.5 years. Of the children who did attend the 4-month consultation (*n* = 142), 86.6% (*n* = 123) were of normal weight status at 3.5 years, and 13.4% (*n* = 19) were overweight. No further analysis could be undertaken.

Similarly, weight status of 3.5-year-old children who did or did not attend the 12-month consultation (healthy eating and active play discussion) were compared. Of the children who did not attend the 12-month consultation, 100% (*n* = 18) were within the normal weight status at 3.5 years. Of the children that did attend the 12-month consultation (*n* = 149), 87.3% (*n* = 130) were of normal weight status at 3.5 years; 12.7% (*n* = 19) were overweight. No further analysis could be undertaken.

### 3.3. Qualitative Results

The following results from parent and nurse interviews are presented per the major themes of the interview schedules (healthy eating, active play and information sources), and reported barriers/enablers to implementing MCH recommendations. This includes emergent themes of ‘relaxed attitudes’ and ‘MCH supportiveness’. Results from the MCH nurse interviews and parent interviews are reported together within each theme.

#### 3.3.1. Healthy Eating

MCH nurses reported discussing child diet from four months to equip parents with the correct knowledge before starting solid foods (“anticipatory guidance”). After the four-month consultation both nurses continued to ask how the child was eating and if they were eating a variety of foods. 

Parents found MCH recommendations were useful for breastfeeding, transition to solid foods, healthy drinks for toddlers and healthy eating for toddlers. Recommendations and advice on transition to solid foods were particularly valued by parents due to the tailored advice to meet the family and individual parent needs. For example: “*for me personally, obviously being the third child, time was an issue.* [MCH nurse] *was really good with thinking outside the box with what was simple and quick- like a banana or avocado*” (P3 interview). Meal times and fussy eating were topics many parents struggled with, and is discussed further within the ‘barriers to implementation’ section.

#### 3.3.2. Active Play

MCH nurses reported promoting activities such as playgroup and organised activities but felt they did not need to encourage parents to be more active with their children as children aged 2–5 are ‘naturally active’. Parents agreed that they did not need to support their child to be physically active due to the large space available on their property and children being involved in farm work and naturally curious. Recommendations and advice on how to implement tummy time were highly regarded by parents due to practical advice given by MCH nurses. For example, “*If your bub was really, really upset, put them on a pillow or put them on the couch and have your face in front of theirs*” (P9 interview). Screen time recommendations of ‘no screen time for 0–2-year old’s’ were reported as difficult to follow for many parents, as discussed within the ‘barriers to implementation’ section. 

#### 3.3.3. Information Sources

Parents were asked to identify their top three sources of information on healthy eating and active play for their children. The MCH service featured in every parent’s top three sources (*n* = 15). Other common information sources included family (*n* = 9), friends (*n* = 7), internet (*n* = 6), information gathered during their studies for their profession as a nurse, teacher, ambulance officer or childcare worker (*n* = 6), and from specialists e.g., dietitian (*n* = 3). 

#### 3.3.4. Enablers to Implementing MCH Recommendations

Interviews with the MCH nurses confirmed the local service has excellent levels of engagement with local families. MCH nurses regularly met with parents in addition to the ten key ‘ages and stages’ consultations. Nurses reported this level of engagement enabled them to build rapport with the family and develop an understanding of the family dynamic and existing parental knowledge. This deepened understanding of the family unit helped MCH nurses to tailor conversations with parents and navigate difficult topics such as decisions on feeding style and addressing overweight children. 

#### 3.3.5. Supportiveness of MCH Service

A high level of engagement and trust in the MCH service was evident through parent interviews. One nurse attributed this to the rurality, lack of other services available and ability of the MCH system to offer additional consultations: “*because of where we live, often we’re the only service they get. We offer additional visits, and they’ll often just take them because they can*” (N1 interview). This high level of engagement was identified as a supportive factor for nurses delivering health messages “*often… I’ve seen the family at least 10 times before the 4 months solids* [discussion]… *So you have an idea already of how their family works… you may have already had the discussion before the 4 month* visit” (N1 interview). 

All but one parent described the MCH service as highly accessible. Parents from smaller towns within the LGA did not feel ‘restricted by time’ during their MCH consultations. Parents gave examples where they had received MCH support via text messages, phone calls or impromptu conversations at the local supermarket. Twelve parents reported seeking additional consultations or informal advice from MCH nurses regarding their child’s health and wellbeing, particularly when their child was ‘really young’. Parents reported feeling they needed the extra guidance in the first 12 months of parenthood, particularly first time parents. “*I just didn’t know where else to go, and she was the first person I thought of speaking to*” (P4 interview). 

#### 3.3.6. Barriers to Implementing MCH Recommendations

There was consensus among the MCH nurses that parents were generally receptive to health promoting messages because “*parents by and large want to do their best for their child*” (N2 interview). Both nurses agreed that parents saw value in the advice and recommendations they provided but acknowledged that there may be barriers to implementing that advice such as financial constraints, food insecurity due to rurality, time constraints associated with being a busy mother or having older children who ‘plead’ for unhealthy foods. 

MCH nurses reported they experienced few barriers to facilitating healthy eating and active play conversations with parents. One nurse described some parents, who had heard the information from MCH consultations for their older children, are not very responsive to lengthy conversations, highlighting the need to tailor advice to meet the parents’ needs. Both MCH nurses reported feeling a level of discomfort when addressing the health behaviours of an overweight child if the mother was also overweight. MCH nurses expressed concern that parents might disengage with their service if these conversations were not appropriately navigated: “*It’s diplomacy, you know, making sure that you don’t offend, but you do get the message across*” (N2 interview). Both MCH nurses found the growth charts to be useful when having these conversations with parents to show weight tracking over time and how recent events may have caused a shift in the child’s weight. “*If they’re crossing lines [on the growth chart] if they are putting on too much weight, we want to know why, what’s happening? … It’s in those instances that we particularly refer to the growth chart so that they can see what’s happened*” (N2 interview).

Overall, parents reported few barriers to implementing the majority of MCH recommendations. Advice on screen time was the only topic parents either did not recall MCH advice or could recall advice but found it difficult to implement at home. Parents who reported struggling to follow screen time advice attributed this to screens being part of adult life: “*If I’m watching something she’ll sit down next to me, not because she’s interested in the screen but because she wants to be with me*” (P15 interview). Parental attitude towards screen time varied significantly with some parents stating they were strongly against large amounts of screen time, and others feeling their children had too much screen time but did not have strategies to prevent this.

Parents appeared to struggle to implement messages regarding meal times and fussy eating. Some parents identified MCH nurses had advised them to avoid watching television and to role model healthy eating behaviours during meal times. Parents recalled MCH nurses asking how their child was eating at the 12-month consultation however difficulties with meal times and fussy eating were not an issue until their child was 2–3 years old. Parents recalled being told to offer the same foods repeatedly however few found this advice had worked. Some parents viewed fussy eating in toddlers as “*normal*” behaviour or “*just a stage*” (P6 interview) therefore did not seek MCH advice. Instead, parents reported an increased use of ‘other’ information sources such as internet, Facebook and friends to combat issues around meal times and fussy eating. 

While parents reported following MCH recommendations diligently for their youngest children, parents showed more relaxed attitudes towards healthy eating/active play for their older children. A parent with four children between the ages of one and ten years old gave the example “*like obviously if you go to McDonalds with a 9-month-old you’re not going to give them that*” (P10 interview). Similarly, attitudes towards healthy eating messages for children and adults was contradictory; “*my husband drinks coke, but that’s completely different, he’s an adult he can make his own decisions*” (P10 interview).

## 4. Discussion

This study aimed to examine the level of childhood obesity in an area known for high rates of adult overweight/obesity, and the extent to which the local MCH support service for parents prevented childhood obesity. The focus was on parental translation of healthy eating and active play MCH recommendations into practice. Local rates of obesity at 3.5 years were significantly lower than the national average (11.38% vs. 20%) (*p* = 0.05) [2]. Parents were highly engaged with the local MCH service with 98% of parents attending the final 3.5-year consultation [22], and the average parent schedules seven additional consultations per child with MCH nurses. Parent interviews showed MCH nurses were a trusted source of information and highly regarded within the community. Parents reported following most MCH recommendations. 

Rates of overweight and obesity at 3.5 years are much lower than national rates, however the upward trend toward obesity starting from 12 months and continuing through to 3.5 years is of concern, particularly as obesity rates of adults 18 years and over in the area are at 63.5% [32]. This increase in obesity is reflective of national trends that show obesity prevalence increasing with age from 20% of 2–4-year-olds to 32.9% at 16–17 years old [2]. The MCH service supports children and parents until the children are 6 years old. Once children are school aged there is limited opportunity for routine weight assessment or discussion with parents about their child’s weight status [33]. The observed relaxation of parent attitudes towards healthy eating and active play behaviour for older siblings and adults may contribute to the increased likelihood of obesity as children age. Interventions targeting primary school aged children (6–12 years old) have been successful in reducing child adiposity through a combination of school based programs and education of staff and parents [15]. Our findings strengthen the argument for a review of the local kindergarten and school systems and how parents can be supported to continue to promote healthy eating and active play for their children after exiting the MCH service. 

Parents in our study struggled to follow screen time recommendations. This issue is not unique to this study. Existing literature shows an estimated 44% of 4–5-year-olds in Australia watch more than the recommended two hours of screen time per day [34]. The concept of screen time has changed significantly since the recommendations in the KAS framework were first printed in 2009, with tablets and smart phones constantly accessible, and increasingly relied on for daily tasks, education and social networking [34,35]. The current recommendations for 0–2 year-olds reads ‘screen time is not recommended for toddlers under two years’ [36]. The recommendations have been updated to define ‘screen time for recreation is not recommended’. However as outlined by Yu and Baxter, more support may be required for parents to manage their children’s screen time and ensure screen time is not prioritised above other activities such as sports, socialising with others that are essential for their healthy development [34]. 

Parents also reported struggling with child fussy eating behaviours, particularly around acceptance of vegetables. Many parents reported experiencing this issue but not seeking support from the MCH service, the explanation given by parents interviewed included “it’s just a stage” or “it’s normal”. It appears parents value MCH support for crucial developmental topics such as tummy time or transition to solids however more non-crucial behaviours such as food acceptance and screen time are not high priority topics for discussion for parents. This may pose an issue if the social and family norm is to accept refusal to eat vegetables and sedentary screen time behaviours [37], particularly as these behaviours can track into adolescence further contributing to the risk of obesity [5]. Factors that influence parental feeding practices include information sources, their own upbringing, practices of other parents around them and existing beliefs [38]. Only 4.1% of adults in the LGA meet the daily requirements for fruit and vegetables consumption [20], therefore low consumption of fruit and vegetables may be the local norm. Changing social norms is a significant health promotion challenge in obesity prevention and it may need to start in early childhood. 

### 4.1. Recommendations

1. To review the Victorian KAS policy and framework, particularly screen time recommendations with the consideration to add strategies for parents to employ to reduce child screen time.
2. To review the obesity prevention support and education offered to parents once their children leave the MCH service and enter local kindergartens and schools.
3. To replicate our study in other LGAs to examine parental translation into practice and relationship to childhood obesity in other settings. Our findings indicate the high level of accessibility of MCH nurses was a key reason for parent uptake of service and advice, this may differ in other areas therefore should be explored.

### 4.2. Strengths

The strengths of this study include the mixed methods approach to triangulate the results. The amount and quality of the data analysed was another significant strength, with access to the entire population of MCH nurses (*n* = 2), population data for the MCH child measurements, retrospective data for the past 5 years; *n* = 438, objectively taken by MCH nurses. Parent interviews were conducted with a representative sample of parents (*n* = 15) based on average annual birth rates (60 births per year) [39]. Additionally, parents were included from the most populous town and smaller more rural towns in the north and south of the LGA. This data enabled us to explore the circumstances unique to parents raising children across the LGA.

### 4.3. Limitations

As we sought to explore childhood obesity within a defined area using a case study approach, the findings of this study are not generalizable to other settings. Additionally, parent information such as education level, household income and parent BMI were not collected but LGA averages indicate, relative to Victorian averages, poorer outcomes on these indicators. Parent self-report information on meal times was collected however objective data such as observing parent role model behaviour or home environment (type of foods offered, television during meal times) were also not collected. Given these factors play a role in parent behaviours and child obesity, our findings should be interpreted with caution.

A sibling effect was described in both parent and nurse interviews where older sibling behaviours had an impact on parents implementing healthy eating/active play recommendations for younger children. We did not examine differences in sibling adiposity as the information was not provided to the researchers, however, this may be worthy of future investigation to quantify concordance of weight status among family members.

All efforts were made to recruit a range of parents and processes were put in place to reduce bias, however social desirability bias may have influenced some responses of parents during interview, particularly because they were reporting on a service they were currently using and the area is a small, close knit community.

## 5. Conclusions

Findings suggest that parents engage strongly with the local MCH service, and translate most of the healthy eating and active play recommendations into practice. Low levels of child overweight/obesity found in the anthropometric data coupled with the data showing parents rate the MCH service as a top information source for healthy eating/active play advice suggests the local MCH service is supporting parents to raise healthy weight children in this area. As this study utilised a case-study design to explore the experiences of nurses/parents locally, the findings are not generalizable to other locations. However, this study adds to the limited knowledge about obesity prevention in early childhood and is the first to describe the experiences of parents in translating early childhood obesity prevention messages into practice. 

## Figures and Tables

**Table 1:** Age, anthropometric measurements and BMI-z of participants at MCH consultations from 2011 until 2016.

Consultations	4 Months		12 Months		18 Months		2 Years		3.5 Years	
*N*	353		320		297		255		174	
	M	SD	M	SD	M	SD	M	SD	M	SD
Age (months)										
Total	4.15	0.49	12.27	0.48	18.42	0.76	24.83	1.68	43.52	1.66
Female	4.13	0.55	12.29	0.53	18.41	0.80	24.99	1.96	43.34	1.28
Male	4.16	0.45	12.26	0.33	18.43	0.73	24.69	1.42	43.68	1.93
Weight (kg)										
Total	6.74	0.83	10.03	1.19	11.66	1.37	13.15	1.56	17.03	2.13
Female	6.37	0.72	9.62	1.06	11.21	1.16	12.86 *	1.66	16.51 *	2.17
Male	7.05	0.79	10.35	1.19	12.00	1.43	13.39	1.44	17.48	2.00
Length (cm)										
Total	62.83	2.61	75.55	2.86	82.64	3.35	88.20	3.65	101.64	3.92
Female	61.96	2.08	74.79	2.70	81.76 *	3.44	88.00	3.62	100.77	3.73
Male	63.52	2.78	76.15	2.85	83.32	3.11	88.40	3.68	102.42	3.95
BMI-z										
Total	0.057	1.34	0.61	1.00	0.76	1.04	0.73	1.00	0.75	1.03
Female	−0.10	0.99	0.50	0.92	0.69	0.96	0.60	0.97	0.59	0.95
Male	0.18	1.56	0.69	1.05	0.80	1.09	0.83	1.01	0.88	1.08
	*N*	%	*N*	%	*N*	%	*N*	%	*N*	%
Weight status—WHO [29]										
Normal										
Total	329	95.36	293	92.72	262	90.34	216	88.89	148	88.62
Female	150	98.04 *	133	95.00	118	93.65	101	94.39	71	89.87
Male	179	93.23	160	90.91	144	87.80	115	84.56	77	87.50
Overweight/Obese										
Total	16	4.64	23	7.28	28	9.66	27	11.11	19	11.38
Female	3	1.96 *	7	5.00	8	6.35	6	5.61 *	8	10.13
Male	13	6.77	16	9.09	20	12.20	21	15.44	11	12.50
Weight status IOTF [30]										
Thinness/Normal										
Total	N/A		N/A		N/A		189	91.30	141	84.94
Female	N/A		N/A		N/A		87	93.55	69	87.34
Male	N/A		N/A		N/A		102	89.47	72	82.76
Overweight/Obese										
Total	N/A		N/A		N/A		18	8.70	25	15.06
Female	N/A		N/A		N/A		6	6.45	10	12.66
Male	N/A		N/A		N/A		12	10.53	15	17.24

* indicates statistically different difference between sexes at *p* < 0.05. Abbreviations: MCH: Maternal Child and Health, *N*: number of children, M: mean, SD: standard deviation, WHO: World Health Organization, IOTF: International Obesity Task Force, BMI-z: body mass index z score.

**Table 2:** Logistic regression model estimating effects of key consultations on weight status.

Variable	OR	*p* Value	95% CI
Consultation			
12 months	Base		
4 months	0.81	0.67	0.29, 2.20
18 months	1.45	0.40	0.60, 3.49
2 years	2.20	0.06	0.96, 5.0
3.5 years	2.38	0.04	1.05, 5.42
(Constant)	0.54	<0.001	0.03, 0.11
Trend	*Z* = 3.93	<0.001	

Abbreviations: OR: odds ratio, CI: confidence interval.

## Appendix A. Interview Guide for Parents

**Breastfeeding**	1. Have you breastfed your baby? If ‘no’—did you formula feed your baby?
	2. Are you currently breastfeeding/formula feeding your baby?
	3. For approximately how long did you breastfeed/formula feed your baby?
	4. How long did you exclusively breastfeed for? *Prompt for child age at which stopped breastfeeding/formula feeding*
	5. Did you use infant formula at any stage? How old was your baby?
**Information sources**	6. Where has most of your advice come from in relation to healthy eating and active play for your child? *Prompt for top three and provide examples:* - MCH Nurse - Doctor - Friends - Family - Internet - Playgroup - Magazine and books - Other
**Food in first year of life** 4-month consult	7. When did your baby first try solid food? *Prompt for child age*
	8. Do you remember what the first food your baby tried was? *Prompt- what food was this? (if Farex prompt if made with breastmilk or water*)
	9. What advice or support did the MCH nurse provide you in in this transition to solids?
	10. Did you find this advice/support useful? In what ways?
	11. What made it harder or easier for you to implement this transition to solids?
**Tummy time** Home visit onwards	12. What advice or support did MCH provide you about ‘tummy time’ for your infant?
	13. Did you find this advice/support useful? In what ways?
	14. What made it harder or easier for you to implement tummy time?
**Screen time** 4-month and 12-month consults	15. What advice/support did MCH provide you about screen time including computers, ipads, and phones?
	16. Was this advice/support useful? In what ways?
	17. What made it harder or easier for you to implement this advice?
**Drinks for children** 4-month consult and 12 month-consult	18. What advice or support did MCH provide you about drinks for your infant?
	19. Was this advice useful? In what ways?
	20. Was it easy for your child to not drink sugary drinks? *Note any response including:* - Fruit juice, cordial, soft drinks, flavoured water - Water is the preferred drink - Breast milk, infant formula, full fat cow’s milk
**Healthy eating for young toddlers** 12-month consult	21. Was your child a fussy eater?
	22. Can you tell me about meal times at your house?
	23. What advice or support did MCH provide to assist you in providing healthy food including fruit and vegetables to your child?
	24. Was this useful? In what ways?
	25. What made it harder or easier for you to implement this advice?
	26. Did you try any other methods to get your child to eat?
	27. What worked for you in introducing your child to fruit and vegetables?
	28. Is your child still a fussy eater? (if applicable)
**Snacks**	29. What do your children usually eat for snacks between meals?
	30. Did this information come from MCH nurses? *Prompt: if ‘no’ where did this information come from?*
**Activity**	31. What activities does your child enjoy?
	32. Does your child play outdoors every day?
	33. How has MCH supported you with these activities?
**Healthy eating and play for kindergarten** 3.5-year consult	34. How has MCH supported you and your child’s eating habits? *Prompt- has MCH supported you around eating meals together, eating according to appetite*
	35. How has MCH supported you to provide your child foods from a range of food groups?
**BMI**	36. Has your MCH nurse discussed your child’s weight status, BMI or developmental tracking with you?
	37. If yes, was this information useful to you?
**Parent support**	38. Have you engaged in any additional MCH visits? *Prompt: for what reasons did you seek additional MCH support? Were these useful to you?*
	39. What support did you have in between MCH visits? *Prompt: family? Friends? Doctor?*
Thank you for participating in this interview. Would you like to receive a copy of the overall report? If yes, please provide email address or other contact details.	

## Appendix B. Interview Guide for Maternal and Child Health Nurses

**Weight status**	1. How often do you discuss with parents their child’s weight status? *Prompt: when would you usually discuss weight status with parents?*
**Healthy eating**	2. How often so you discuss with parents their child’s diet and healthy eating? *Prompt: when would you usually discuss child healthy eating with parents?* *Prompt: what would you usually discuss?*
**Active play**	3. How often do you discuss with parents the importance of active play for their children? *Prompt: when would you usually discuss active play with parents?* *Prompt: what would you usually discuss?*
**Barriers**	4. Are there any barriers to you discussing children’s healthy weight status, healthy eating and active play? If ‘yes’*: could you please describe these generally?*
	5. In your experience, how do parents receive these messages?
**Key Ages and Stages**	6. Do you find the resources within the Ages for Stages framework helpful during discussions relating to healthy weight status, healthy eating and active play?
**Implementing advice**	7. Do you feel parents follow your advice on healthy eating and active play? *Prompt: why? What supports this?*
	8. Do you think the internet plays a role in this?
	9. Do you feel parents sometimes do not follow your advice on healthy eating and active play? *Prompt: why? What are the barriers?*
**Additional consultations**	10. What do parents usually seek advice on during ‘additional’ visits to MCH? *Prompt: could you narrow these down to a top 3?*
**General**	11. Do you think there is enough support in Yarriambiack for parents to raise healthy weight children?
